# Feasibility and effects of using location‐tracking devices to individuals living with dementia: An integrative review

**DOI:** 10.1002/alz.088638

**Published:** 2025-01-09

**Authors:** Jayeong Kim, Mina Hwang, Yeji Hwang

**Affiliations:** ^1^ Seoul National University, Seoul, Seoul Korea, Republic of (South)

## Abstract

**Background:**

Wandering is a common issue among individuals living with dementia. This behavior often leads to increased worry for family caregivers, as those with dementia are at a heightened risk of becoming lost. Location‐tracking devices have potential as effective tools for providing independence to individuals living with dementia while reducing the risk of them getting lost. However, there is limited knowledge regarding the effectiveness of these devices specifically for people living with dementia. This study aims to examine the feasibility and effects of using location‐tracking devices in individuals living with dementia.

**Methods:**

An integrative review was conducted using five databases: PubMed, Embase, Web of Science, CINAHL, and Scopus. Literature published up to August 2023 was screened for review. The inclusion criteria for a full‐text review were as follows: Empirical studies which (1) applied location‐tracking devices, (2) explored the effects of applying these devices, (3) involved a population of individuals living with dementia or their caregivers, and (4) were published in peer‐reviewed journals. Among the 1,429 studies initially identified, 10 studies were ultimately included in the review.

**Results:**

The severity of cognitive impairment in the study participants varied across the studies. The duration of using the location‐tracking devices also varied from 1 day to 3 years. The location‐tracking devices were either worn on a belt, on the wrist as a watch, carried in a pocket, or embedded in mobile phones. Overall, these devices proved beneficial for both individuals living with dementia and their family caregivers. For individuals living with dementia, the devices provided greater independence when going outside and enhanced their sense of safety. For caregivers of individuals living with dementia, the devices alleviated concerns about their loved ones getting lost.

**Conclusions:**

Location‐tracking devices have the potential to increase independence in individuals living with dementia and to lessen psychological stress among family caregivers. Researchers and stakeholders need to put more effort into encouraging the use of location‐tracking devices and minimizing the barriers.

